# Timing of Coronavirus Disease 2019 (COVID-19) Vaccination and Effects on Menstrual Cycle Changes

**DOI:** 10.1097/AOG.0000000000005550

**Published:** 2024-02-27

**Authors:** Alison Edelman, Emily R. Boniface, Victoria Male, Sharon Cameron, Eleonora Benhar, Leo Han, Kristen A. Matteson, Agathe van Lamsweerde, Jack T. Pearson, Blair G. Darney

**Affiliations:** Department of Obstetrics and Gynecology, Oregon Health & Science University, and Oregon Health & Science University-Portland State University, School of Public Health, Portland, Oregon; the Department of Metabolism Digestion and Reproduction, Imperial College London, London, United Kingdom; Obstetrics and Gynaecology, University of Edinburgh and Chalmers Centre, Edinburgh, Scotland; Natural Cycles USA Corp, New York, New York; the Department of Obstetrics and Gynecology, University of Massachusetts Chan Medical School, Worcester, Massachusetts; and the National Institute of Public Health, Center for Population Health Research, Cuernavaca, Mexico.

## Abstract

**OBJECTIVE:**

To assess whether menstrual cycle timing (follicular or luteal phase) of coronavirus disease 2019 (COVID-19) vaccine administration is associated with cycle length changes.

**METHODS:**

We used prospectively collected (2021–2022) menstrual cycle tracking data from 19,497 reproductive-aged users of the application “Natural Cycles.” We identified whether vaccine was delivered in the follicular or luteal phase and also included an unvaccinated control group. Our primary outcome was the adjusted within-individual change in cycle length (in days) from the average of the three menstrual cycles before the first vaccination cycle (individuals in the unvaccinated control group were assigned a notional vaccine date). We also assessed cycle length changes in the second vaccination cycle and whether a clinically significant change in cycle length (8 days or more) occurred in either cycle.

**RESULTS:**

Most individuals were younger than age 35 years (80.1%) and from North America (28.6%), continental Europe (33.5%), or the United Kingdom (31.7%). In the vaccinated group, the majority received an mRNA vaccine (63.8% of the full sample). Individuals vaccinated in the follicular phase experienced an average 1-day longer adjusted cycle length with a first or second dose of COVID-19 vaccine compared with their prevaccination average (first dose: 1.00 day [98.75% CI, 0.88–1.13], second dose: 1.11 days [98.75% CI, 0.93–1.29]); those vaccinated in the luteal phase and those in the unvaccinated control group experienced no change in cycle length (respectively, first dose: −0.09 days [98.75% CI, −0.26 to 0.07], second dose: 0.06 days [98.75% CI, −0.16 to 0.29], unvaccinated notional first dose: 0.08 days [98.75% CI, −0.10 to 0.27], second dose: 0.17 days [98.75% CI, −0.04 to 0.38]). Those vaccinated during the follicular phase were also more likely to experience a clinically significant change in cycle length (8 days or more; first dose: 6.8%) than those vaccinated in the luteal phase or unvaccinated (3.3% and 5.0%, respectively; *P*<.001).

**CONCLUSION:**

COVID-19 vaccine–related cycle length increases are associated with receipt of vaccination in the first half of the menstrual cycle (follicular phase).

## Abstract 2

Individuals receiving the coronavirus disease 2019 (COVID-19) vaccination during the follicular phase were more likely to experience menstrual cycle length changes than those receiving vaccine in the luteal phase.

We now have a body of evidence demonstrating that the coronavirus disease 2019 (COVID-19) vaccine is associated with temporary menstrual cycle disturbances at the population level.^1–4^ The underlying mechanism for a vaccine-related cycle length disturbance is still under investigation. The leading hypothesis is that these disturbances are due to the immune response that vaccines are designed to produce. The hypothalamic-pituitary-ovarian (HPO) axis is responsible for the cascade of events that regulates the timing of the menstrual cycle, and the immune and reproductive systems interact closely with one another.^5^ The menstrual cycle is driven by hormonal signaling through the HPO axis, and a variety of stressors are known to act on this axis, resulting in increased cycle length, particularly when the stressor acts in the follicular phase.^6,7^ Systemic immune responses may act as one such stressor, and it is well-established that cytokines, produced as an early event in the vaccine response,^8,9^ can directly affect the HPO axis,^10,11^ suggesting that timing of COVID-19 vaccination may be the driver of the observed changes in cycle length.

A known ovulation date and overall menstrual cycle length is the most accurate way to calculate the duration of an individual's follicular and luteal phase, but this requires invasive and serial sampling of blood or urine, sometimes in combination with ultrasound monitoring of the ovary. However, given the stability of luteal phase duration, menstrual cycle tracking with or without basal body temperature documentation is used extensively as a proxy to divide the menstrual cycle into the follicular and luteal phases. In this study, we leveraged prospectively collected menstrual cycle tracking data from individuals using a digital fertility-awareness application with a validated algorithm predicting ovulation date to improve on prior research that approached this question but had insufficient sample size to determine whether an effect exists, no control group, or no validated ovulation date.^12–14^

In this retrospective cohort analysis, we used menstrual cycle data to determine whether timing of COVID-19 vaccination (vaccination in the follicular phase vs the luteal phase) is associated with changes in menstrual cycle length. We compared three groups: individuals vaccinated in the follicular phase, individuals vaccinated in the luteal phase, and an unvaccinated control group. We also assessed how the proportion of individuals who experience clinically significant changes in menstrual cycle length after vaccination differs based on the menstrual phase of vaccination receipt.

## METHODS

We conducted a retrospective cohort analysis of menstrual cycle data from the Natural Cycles digital application. Individuals prospectively track their menstrual cycles using Natural Cycles to prevent or plan pregnancy without the use of hormonal contraception methods and may consent to use of their deidentified data for research purposes or withdraw consent if requested. This study protocol was approved by the Oregon Health & Science University IRB and the Reading Independent Ethics Committee, United Kingdom. Details of all information tracked by the application have been described elsewhere.^15^ To be eligible for this study, individuals must have consented to use of their data for research purposes, recorded at least one complete cycle after October 1, 2020, and provided details about their COVID-19 vaccination status. Additionally, individuals had to be aged 18–45 years; at least three cycles postpregnancy, post–positive pregnancy test, or post–hormonal contraception use for the full study period; with normal menstrual cycle length (24–38 days, as defined by the International Federation of Gynecology and Obstetrics)^16^ before vaccination; with known geographic location; and not menopausal. Individuals also needed to be vaccinated within the first 38 days of the menstrual cycle to exclude those already experiencing an abnormally long cycle before vaccine exposure.

Menstrual cycle data ranged from October 1, 2020, to November 7, 2021, and initial COVID-19 vaccines were received between January 2 and October 31, 2021. Each individual contributed data from a minimum of four consecutive cycles. For vaccinated individuals, we included the three prevaccination cycles and at least the cycle in which the first vaccine dose was received. If available, we also included cycles through the second vaccine dose cycle. For unvaccinated individuals, we included four to five consecutive cycles from a similar time period, depending on the availability of data from the fifth cycle. If data were not available for the second vaccine dose cycle (vaccinated) and fifth consecutive cycle (unvaccinated), we excluded those individuals from analyses related to the second dose. The analytical sample for this study represents a subset of our previous work,^1^ excluding any individuals who did not receive their first vaccine dose within the first 38 days of their cycle (n=125).

Our three-category primary exposure was timing of COVID-19 vaccination, comparing unvaccinated individuals with those who received the vaccine in the follicular phase and those who received it in the luteal phase. Individuals reported their vaccination dates or confirmed their unvaccinated status using an in-application message. Natural Cycles uses a proprietary algorithm based on long-standing evidence that uses daily basal body temperature to identify an individual's ovulation date for each cycle and, if logged, urine LH test results, which we used to determine the phase in which a vaccine was received: follicular phase (cycle day 1 through ovulation date) and luteal phase (day immediately after ovulation through the end of cycle). In cycles in which basal body temperature data were inadequate to determine the ovulation date using the algorithm (approximately 20% of vaccination cycles), we categorized the luteal phase as the final 14 days of the cycle and the follicular phase as preceding that and performed additional sensitivity analyses (see below).^16^

Our primary outcome was the adjusted within-individual change in cycle length (in days) from the average of the three prevaccination cycles to the first vaccination cycle. For the vaccinated cohorts (both follicular and luteal phase), cycle four was the first vaccination cycle; the second vaccination cycle varied depending on whether and when the second dose was received (cycles 4–13, with cycle five being most common). For the unvaccinated cohort, we designated cycles four and five as the notional first and second vaccination cycles, respectively, and cycles one through three as the equivalent of the prevaccination cycles. We considered three secondary outcomes: the adjusted within-individual change in cycle length from the prevaccination average to the second vaccination cycle and the proportion of individuals who experienced a *clinically significant change from their prevaccination average*, defined as an increase or decrease of 8 days or more,^16^ during the first and the second vaccination cycles. We were not able to assess changes from the three cycles immediately before the second dose to the second vaccination cycle due to the very small number of individuals with three cycles between their first and second dose (n=121, 0.9% of sample).

We included additional sociodemographic characteristics collected by Natural Cycles. We categorized age into roughly 5-year groups: 18–24, 25–29, 30–34, 35–39, and 40–45 years. Race and ethnicity were reported as East or Southeast or South or Central Asian (combined into a single Asian category due to sample size), Black or African or African American, Hispanic or Latina, Middle Eastern or North African, Native Hawaiian or Pacific Islander, or White. We included race in the study to describe our sample, acknowledging that race is not associated with menstrual health. We categorized body mass index (BMI, calculated as weight in kilograms divided by height in meters squared) as underweight (lower than 18.5), normal weight (18.5–24.9), overweight (25.0–29.9), and obesity (30.0 or higher). We classified country of residence into geographic regions: United Kingdom and Channels Islands, continental Europe, United States and Canada, or all other regions. The majority of individuals in the continental Europe category lived in Sweden (55.7%), and the majority in the other regions category lived in Brazil (62.0%). We also included parity (nulliparous vs parous), education (at least a college degree vs less education), and relationship status (in a relationship vs not). Finally, we included the vaccine mechanism of action and brand for those in the vaccinated groups: mRNA (Moderna, Pfizer), adenovirus vector (Astrazeneca, Covishield, Johnson & Johnson, Sputnik), inactivated virus (Covaxin, Sinopharm, Sinovac), and unspecified. Due to differences in data collected over time and country-specific regulations on data collection, most variables had a large degree of missing data.

We compared sociodemographic characteristics of the sample by vaccination timing groups: follicular phase, luteal phase, and unvaccinated. To calculate the adjusted differences in cycle length, we first used multiple imputation by chained equations with 50 imputed data sets due to the large amount of missing sociodemographic characteristics. We then used the imputed data sets to create linear regression models for both the first and second vaccination cycles. For each model, the dependent variable was the change in cycle length, the primary independent variable was the vaccination timing group, and models were adjusted for age, race and ethnicity, parity, BMI, education, relationship status, and geographic region. We then calculated the adjusted change in cycle length for each group predicted by the model and created scatter plots of these estimates. We did not include vaccine type in the adjusted models due to the small sample sizes of several types, the lack of variability within the unvaccinated group, and the fact that none of our previous work has identified differences by vaccine type or mechanism of action.^1–3^

We also assessed crude changes in cycle length, which were similar to the adjusted changes reported here (see Appendix 1, available online at http://links.lww.com/AOG/D603), and created overlaid histograms of the unadjusted changes for the follicular and luteal vaccination groups to compare overall distributions. We performed pairwise comparisons of the proportion of individuals who experienced a clinically significant change in each of the groups (follicular phase, luteal phase, and unvaccinated) for both the first and second vaccination cycles and compared all sociodemographic characteristics by experience of a clinically significant change in cycle length, regardless of vaccination timing or status, to determine whether any factors increased the risk of menstrual disturbances.

All statistical tests were two-sided. We used a Bonferroni-adjusted significance level of 0.0125 for all analyses to accommodate the multiple statistical tests performed for our four outcomes and incorporated an additional Bonferroni adjustment for the three pairwise comparisons of clinically significant changes. All regression estimates are accompanied by 98.75% CIs, and χ^2^ *P*-values have been multiplied by 12 so that the reported values are significant at the 0.05 level. All analyses were conducted in Stata 17.0.

We conducted several sensitivity analyses to determine the robustness of our results. First, we excluded individuals without a known ovulation date using the Natural Cycles algorithm (first dose, n=3,683; second dose, n=2,155). Second, we excluded individuals with any prevaccination cycles outside the 24- to 38-day range (first dose, n=2,951; second dose, n=2,082). Third, we excluded individuals with any prevaccination cycles identified as anovulatory by the Natural Cycles algorithm (first dose, n=874; second dose, n=607 second dose. Fourth, we excluded individuals with a prevaccination follicular phase length outside the 8- to 21-day range (first dose, n=4,822; second dose, n=3,443). Fifth, we excluded individuals who recorded a diagnosis of polycystic ovarian syndrome or who had a prevaccination average cycle length of 36–38 days (first dose, n=919; second dose, n=661). Sixth, we excluded individuals who had recorded any emergency contraception use during the vaccination cycle or any prior cycle (first dose, n=508; second dose, n=481). Finally, we divided both the follicular and luteal phases into early and late subphases of equal length (ie, in the first or second half of the individual's follicular or luteal phase) and compared cycle length changes based on vaccination timing for these five groups: early follicular, late follicular, early luteal, late luteal, and unvaccinated.

## RESULTS

**Fig. 1.**
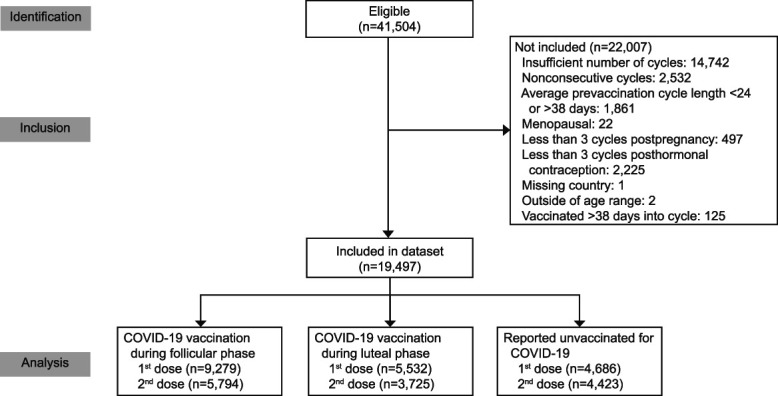
STROBE (Strengthening the Reporting of Observational Studies in Epidemiology) flow diagram. Sample sizes for first and second dose refer to the number of included individuals with cycle data from each dose of the initial coronavirus disease 2019 (COVID-19) dosing regimen. Unvaccinated individuals were assigned a notional vaccination date.

Of 41,504 eligible individuals using the Natural Cycles application, 19,497 met the inclusion criteria (Fig. 1). The final sample included 9,279 individuals who received their first vaccine dose in the follicular phase, 5,532 who received their first dose in the luteal phase, and 4,686 individuals who were unvaccinated (Table 1). The majority of individuals received an mRNA vaccine (63.8%), were younger than age 35 years (80.1%), and were from the United States or Canada (28.6%), continental Europe (33.5%) or the United Kingdom (31.7%). Characteristics of vaccinated individuals did not differ by timing of vaccination. However, unvaccinated individuals were more likely to be younger than age 25 years (19.4% vs approximately 10% for both vaccinated groups), have less than a college degree (25.9% vs approximately 15%), and be from the United States or Canada (42.7% vs approximately 24%).

*Edelman. Menstrual Phase Timing of COVID-19 Vaccine. Obstet Gynecol 2024*.

**Table 1.**
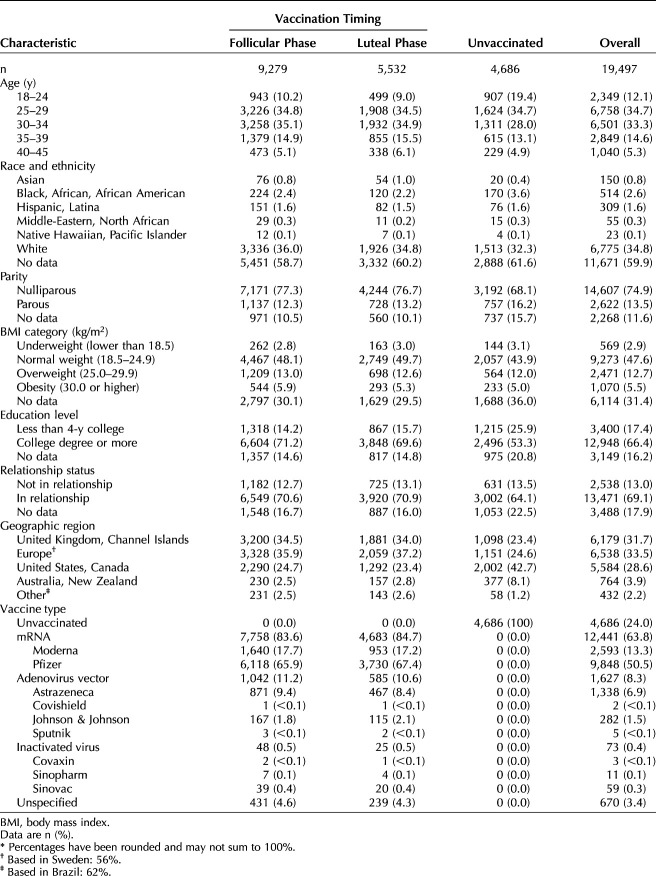
Characteristics of Study Participants, Overall and by Timing of Coronavirus Disease 2019 (COVID-19) Vaccination (N=19,497)*

**Fig. 2.**
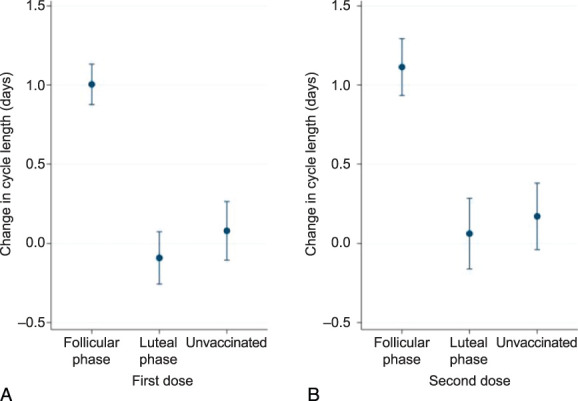
Adjusted within-individual change in menstrual cycle length (in days) from the three-prevaccination cycle average to vaccination cycle, by timing of vaccination, for first **(A)** and second **(B)** coronavirus disease 2019 (COVID-19) vaccine doses. Estimates are adjusted for age group, body mass index category, race and ethnicity, parity, education, relationship status, and geographic region after multiple imputation of missing data. *Error bars* represent 98.75% CIs.

Individuals who were vaccinated in the follicular phase had a 1.00-day change (increase) in cycle length (98.75% CI, 0.88–1.13) from the three prevaccination cycle average to the first vaccination cycle (Table 2; Fig. 2A), after adjusting for age group, BMI category, race and ethnicity, parity, education, relationship status, and geographic region after multiple imputation of missing data. No changes in cycle length occurred for individuals vaccinated in the luteal phase (−0.09-day change, 98.75% CI, −0.26 to 0.07) or for unvaccinated individuals (0.08-day change, 98.75% CI, −0.10 to 0.27).

**Table 2.**
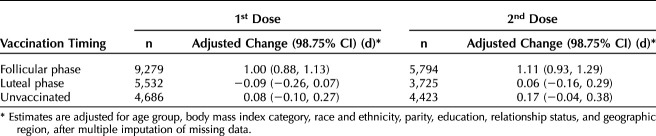
Adjusted Within-Individual Change in Menstrual Cycle Length From the Three Prevaccination Cycle Average to Vaccination Cycle, by Timing of Vaccination, for First and Second Coronavirus Disease 2019 (COVID-19) Vaccine Doses

*Edelman. Menstrual Phase Timing of COVID-19 Vaccine. Obstet Gynecol 2024*.

Results were similar among individuals who received a second vaccine dose (Table 2; Fig. 2B). Individuals vaccinated in the follicular phase experienced an adjusted increase of 1.11 days (98.75% CI, 0.93–1.29) in their second vaccination cycle, whereas those vaccinated in the luteal phase and the unvaccinated experienced no change in cycle length (luteal phase: 0.06-day change [98.75% CI, −0.16 to 0.29]; unvaccinated: 0.17-day change [98.75% CI, −0.04 to 0.38]). Unadjusted results for both the first and second vaccination dose were also similar (Appendix 1, http://links.lww.com/AOG/D603); the distribution for individuals vaccinated in the follicular phase was more right-skewed, with longer tails than for those vaccinated in the luteal phase for both doses (Appendix 2, available online at http://links.lww.com/AOG/D603).

For both the first and second vaccination doses, the percentage of individuals who experienced a clinically significant change in cycle length (8 days or more) was higher for individuals who were vaccinated in the follicular phase than for those who were vaccinated in the luteal phase or were unvaccinated (Table 3). In the first vaccination cycle, 6.8% of people vaccinated in the follicular phase had a clinically significant change (8 days or more), compared with 3.3% of those vaccinated in the luteal phase and 5.0% of the unvaccinated cohort (*P*<.001 for each pairwise comparison). In the second vaccination cycle, again 6.8% of those vaccinated in follicular phase had a clinically significant change in cycle length (8 days or more), compared with 4.7% of those vaccinated in luteal phase and 5.0% of the unvaccinated group. The percentage who experienced a clinically significant change in cycle length in the follicular group was significantly higher than both the luteal and unvaccinated groups (*P*<.001 and .001, respectively), but the luteal and unvaccinated groups were not statistically different from each other (*P*=1.000). Younger individuals (ages 18–29) were more likely to experience a clinically significant change in cycle length for both doses (*P*<.001 both doses).

**Table 3.**
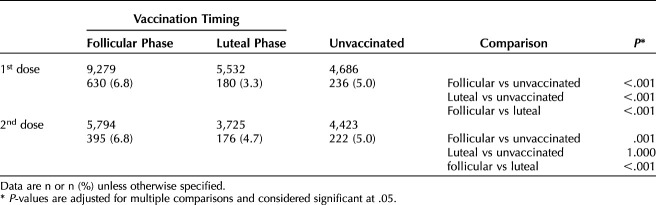
Proportion of Individuals Who Experienced a Clinically Significant Change in Cycle Length (8 Days or More) From the Three Prevaccination Cycle Average to the Cycles Associated With the First and Second Coronavirus Disease 2019 (COVID-19) Vaccine Doses, by Timing of Vaccination

Trends remained consistent across all of our sensitivity analyses: individuals vaccinated in the follicular phase experienced significant increases in cycle length compared with those vaccinated in the luteal phase and the unvaccinated, who were not statistically different from each other. See supplemental appendices (available online at http://links.lww.com/AOG/D603) for results excluding individuals without a known ovulation date (Appendix 3), with prevaccination cycles outside the normal 24- to 38-day range (Appendix 4), with an anovulatory prevaccination cycle (Appendix 5), with a prevaccination follicular phase length outside the 8- to 21-day range (Appendix 6), with polycystic ovarian syndrome or with prevaccination cycle length longer than 35 days (Appendix 7), with reported emergency contraception use in the vaccination cycles or any prior cycles (Appendix 8), and comparing results for early- and late-follicular and luteal phases (Appendix 9). Full modeling results can be found in Appendix 10. See Appendices 11 and 12 for collated results of Appendices 1–9, all available online at http://links.lww.com/AOG/D603.

## DISCUSSION

Our initial studies were able to establish the first association between COVID-19 vaccination and menstrual cycle length changes.^1,2^ Here, we build on those findings to further refine our understanding of how timing of COVID-19 vaccination is associated with this change. These results provide clear evidence that population-level cycle length changes are associated with receipt of the COVID-19 vaccine, specifically during the follicular phase of the menstrual cycle.

Menstrual cycle timing variability results from a cascade of events involved in recruitment of a dominant follicle during the follicular phase, which is regulated by the HPO axis. The HPO axis is sensitive to a variety of “stressors”; if a stressor occurs in the follicular phase, cycle timing can be affected temporarily.^6,7^ The follicular phase is inherently more variable in duration between and within individuals than the luteal phase; thus, variability in menstrual cycle length is derived primarily from the follicular phase.^17^ Vaccines are designed to elicit an immune response. As such, the leading hypothesis for how the COVID-19 vaccine caused temporary cycle length changes was an interaction between the immune and reproductive systems in the follicular phase.^18^ A vaccine given during the follicular phase could extend the cycle by disrupting timing of the luteinizing hormone surge. Luteal phase length is not HPO-dependent; therefore, vaccinations during this phase would not alter when menstruation occurs. Our results are consistent with this mechanistic hypothesis, clearly showing that overall cycle length changes are driven by vaccination in the follicular phase compared with vaccination in the luteal phase or an unvaccinated control group.

Menstrual cycles are inherently variable, and there is the potential for bias in our results if individuals experience a longer menstrual cycle phase in the vaccination cycle for reasons unrelated to the vaccine. We have attempted to mitigate this potential for bias by excluding individuals with evidence of irregular cycling before vaccination and those who already were experiencing a cycle outside the normal range before they received their vaccine. Although it may not be possible to completely eliminate all length-time bias given the innate variability of menstrual cycles, we believe that our findings reflect a true association between timing of vaccination and small increases in cycle length for several reasons.

First, our results remain consistent across a range of sensitivity analyses aimed at eliminating underlying cycle length variability. Second, although the length of the follicular phase is inherently more variable than that of the luteal phase, the luteal phase is also variable and subject to the same issues of bias. If our results were solely driven by length bias, we would expect to see similar, albeit likely smaller, effects in the luteal phase. Instead, we consistently observe no change in cycle length for individuals vaccinated in the luteal phase, even after excluding individuals with unknown ovulation dates, whose luteal phase lengths were imputed as the last 14 days of the cycle (Appendix 3, http://links.lww.com/AOG/D603). Indeed, the point estimates for individuals vaccinated in the luteal phase are consistently lower than for the unvaccinated cohort, although not statistically different. Third, biased results would predict longer changes in cycle length for individuals vaccinated later in their follicular phase. However, we see no significant differences between individuals vaccinated in the first half of their follicular phase and those vaccinated in the second half (Appendix 9, http://links.lww.com/AOG/D603). Fourth, in other work focused on menstrual bleeding, we identified an increase in overall bleeding quantity among vaccinated individuals compared with those who were unvaccinated, with no accompanying change in menses length, a finding that is not subject to the same bias issues.^3^ Finally, global reports of menstrual disturbances after COVID-19 vaccination are numerous.^18–20^ We hope that this study validates those reports using rigorous methodology while reassuring the public that the observed population-level changes are small and not clinically significant for most people.

Strengths of our study include a large global sample, prospectively collected menstrual cycle data, integration of a validated algorithm to identify follicular and luteal phases and date of ovulation, and inclusion of an unvaccinated control group. Our findings are consistent with earlier reports in the literature.^13,14^

Our study also has limitations. First, our findings may not be generalizable to a global population. Natural Cycles users are not using hormonal contraception by design and are more likely to be White, college educated, to live in North America or Europe, and to have lower BMIs than global distributions.^15^ Although geographic location, race and ethnicity, and education are not factors that influence menstrual cycle regulation, an individual's weight and BMI can affect menstrual cyclicity.^6,15^ Our cohort, no matter their BMI, were eligible only if they demonstrated regular cyclicity, and our results are adjusted for BMI category after multiple imputation for missing values. Hormonal contraception inherently controls the regulatory processes involved in menstrual cyclicity, but, again, we included only individuals with proven cyclicity to determine an effect of the exposure.

Second, we do not have data on severe acute respiratory syndrome coronavirus 2 (SARS-CoV-2) infection, which also produces inflammatory responses and likely also disrupts menstrual cycles.^21–24^ However, our data set was from earlier in the pandemic, when infection rates were lower, and our unvaccinated control group, who would be more likely to experience infection, demonstrated no changes in cycle length. Finally, our data are not able to tell us whether the magnitude of immune response or certain aspects of an individual's immune response (innate vs adaptive) are more likely to result in a cycle length disturbance or whether the experience of a cycle length change is indicative of an improved immune response.

Although the population-level effect of the COVID-19 vaccine on cycle length is small, we would offer that the significance of our studies is not the magnitude of change. Many individuals in our sample, particularly those who were vaccinated in the follicular phase, experienced postvaccination changes in cycle length that did not rise to the level of clinical significance but might still cause concern because they were unexpected. No matter the magnitude of change, any unanticipated change in a routine bodily function linked to fertility after a new medical intervention could fuel vaccine hesitancy, leading to long-lasting effects, and it is important to determine why this change is occurring.^25,26^ For example, in Japan, the lack of human papillomavirus vaccine uptake due to vaccine hesitancy is estimated to result in approximately 10,000 preventable deaths from cervical cancer over the next 50 years.^27^

Additional work is also needed to establish whether observed differences vary in key subpopulations, because our data set is focused on data from individuals with regular menstrual cycles prevaccination, and whether any other aspects of the menstrual cycle are affected (eg, menstrual-related symptoms, menstrual flow). Nonetheless, our findings are reassuring in their consistency with long-standing evidence that an acute biological stressor occurring in the follicular phase can temporarily change cyclicity.

## Figures and Tables

**Figure:**
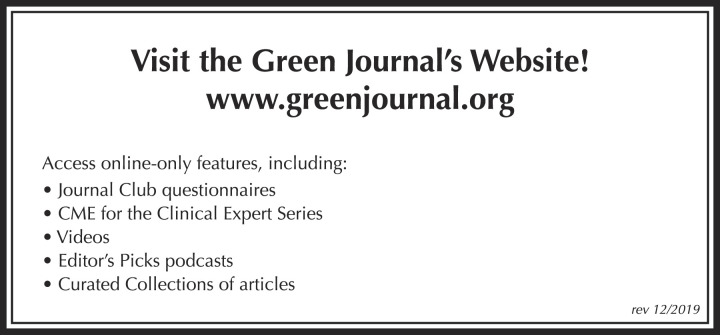
No available caption
